# Supplementary material for: Maternal transmission as a microbial symbiont sieve, and the absence of lactation in male mammals
Source: Nat Commun. 2024 Jun 27;15:5341. doi: 10.1038/s41467-024-49559-5 (PMC11211401; doi:10.1038/s41467-024-49559-5)
Supplement: Supplementary file 1 — Supplementary Information [file 41467_2024_49559_MOESM1_ESM.pdf]

Supplementary Information: Maternal transmission as a microbial symbiont sieve, and the absence of lactation in male mammals

Brennen T. Fagan*
Leverhulme Centre for Anthropocene Biodiversity, University of York and Department of Mathematics, University of York

George W. A. Constable and Richard Law
Department of Mathematics, University of York

Supplementary Methods 1. MODELLING THE BENEFITS OF BIPARENTAL LACTATION

In the main body of the paper, we have focused on a cost to male lactation that is not currently accounted for in the literature, namely that of biparental transmission of deleterious symbionts. This is because we are particularly interested in cases where the benefits of biparental care appear sufficiently high that such care is selected for (as with Azara's owl monkeys), and yet male lactation is not. However accounting for the benefits of male lactation to progeny is also an important consideration.

In order to connect the model more directly to the underlying biology, we introduce two new parameters for the volume of milk produced by mothers, $V^{♀}$, and the volume of milk produced by fathers, $V^{♂}$, for infants. For simplicity, we fix each of these parameters to between 0 (no lactation) and 1 (lactation at some maximum capacity). Simple benefits to biparental lactation are then relatively straightforward to incorporate within our modelling framework.

Drawing on Kokko and Jennions (2008), we introduce an additional survival probability on infants that is a function of the care received from both parents. For simplicity, we equate this care directly with the total volume of milk received from both parents, $V_{\mathrm{Tot}} = V^{♀} + V^{♂}$. For strictly maternal lactation we then have $V^{♀} > 0$ and $V^{♂} = 0$, while for maternal lactation with some paternal lactation we have $V^{♀} > 0$ and $V^{♂} > 0$. The offspring survival probability is given by

$$\Gamma\left(V_{\mathrm{Tot}}\right) = \exp\left[-\gamma/V_{\mathrm{Tot}}\right] \tag{1}$$

$$= \exp\left[-\gamma/(V^{♀} + V^{♂})\right], \tag{2}$$

where decreasing γ decreases the relative benefits of male lactation (see Figure 1, panel (a)).

Having described how offspring survival probability varies as a function of parental lactation, we must now similarly define how vertical symbiote transmission varies as a function of parental lactation. Recall that for a community S that contains an additional symbiont relative to community s, the probability of vertical transmission from a mother is α and from a father is β (see Main Text, Table 1.). We now wish to make these probabilities functions of the volume of milk transmitted from each parent. We choose the functions

$$\alpha(V^{♀}) = \frac{1 - \exp(-\sigma V^{♀})}{1 - \exp(-\sigma)} \tag{3}$$

$$\beta(V^{♂}) = \frac{1 - \exp(-\sigma V^{♂})}{1 - \exp(-\sigma)} \tag{4}$$

as illustrated in Figure 1, panel (b). As the parameter σ tends to zero, we see a linear relationship between the probability of transmitting S from a parent carrying S and the volume of milk supplied by that parent. However $\sigma \gg 0$ captures the more biologically relevant scenario in which only a small inoculum of the symbiote (which can be transmitted from small volumes of parental milk) is necessary for the symbiote to establish in the offspring and thus for community S to be transmitted.

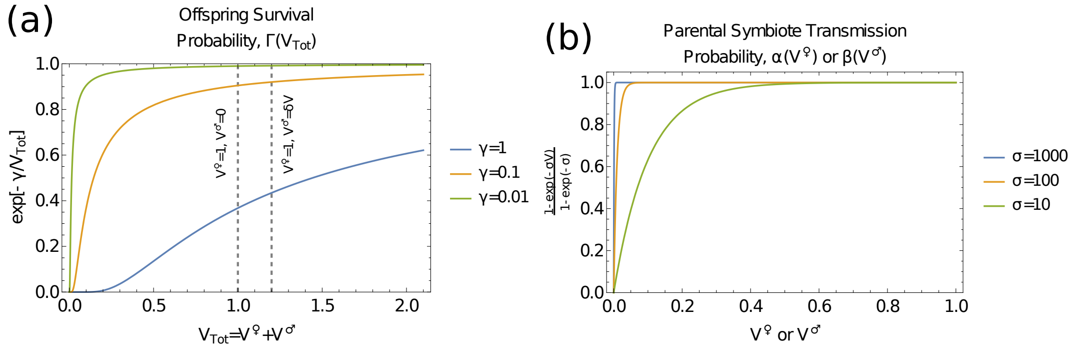

Supplementary Figure 1. Offspring survival and symbiote transmission probability as a function of milk produced by parents. Panel (a): A model for saturating benefits off increased milk supply to offspring (see Eq. (1)). Under strictly maternal lactation ($V^{♀} > 0$ and $V^{♂} = 0$) the survival probability of offspring is given by $\Gamma\left(V^{♀}\right)$ (see leftmost vertical dashed line for $V^{♀} = 1$). Conversely under biparental lactation ($V^{♀} > 0$ and $V^{♂} > 0$) the survival probability of offspring is given by $\Gamma\left(V^{♀} + V^{♂}\right)$ (see rightmost vertical dashed line for $V^{♀} = 1$, $V^{♂} > 0$). The additional survival probability gained from male lactation is then given by $\exp\left[-\gamma/(V^{♀} + V^{♂})\right] - \exp\left[-\gamma/V^{♀}\right]$, which increases with γ. The parameter γ can therefore be understood as controlling the benefits of male lactation to offspring, such as those arising from increased caloric intake. Panel (b): Transmission probabilities of community S from mothers, $\alpha(V^{♀})$, and fathers, $\beta(V^{♂})$, as a function of the milk supplied by either parent ($V^{♀}$ and $V^{♂}$ respectively), as illustrated mathematically in Eqs. (3-4).

A. Exploring the invasion potential of lactating males in a resident non-lactating male population

In order to explore how the benefit of biparental lactation to offspring affects our mathematical model, we use a modified version of the *Differential equation model* in the main text. As with that model, we account for both the density of female hosts x^+ with the symbiont community S, and the density x^- with the community s. However we now have that the transmission probabilities α and β are functions of the volume of milk produced by parents. In addition, we must also now introduce the density of non-lactating (uniparental, $V^{♂} = 0$) male hosts y^+ (with the symbiont community S) and y^- (with the community s) as well as the density of lactating (biparental, $V^{♂} > 0$) male hosts z^+ (with the symbiont community S) and z^- (with the community s). Note that under Fisherian 1:1 sex ratios, the dimensionality of this problem can be reduced by assuming $x^+ = y^+ + z^+$ and $x^- = y^- + z^-$, however we retain the full list of variables here for notational clarity.

As we have seen in the section *Horizontal transmission and the symbiont sieve* of the main text, strict introduction of the symbiont can lead to fixation of the symbiont (see main text, Figure 5). Such elimination of variation in the microbiome community composition of the population also removes the possibility of selection on the modifier gene, M^-, that suppresses lactation in males. Moreover, over long evolutionary time we are not interested in such situations, which correspond to the biologically unrealistic scenario of a uniform microbiome across the entire host population. For this reason, we amend the model in the main text (see Eqs. (5-6) and Eqs. (7-12)) to also allow the possibility of the symbiont being lost. At rate e_0 a symbiont is introduced to hosts in the population (such that community S displaces community s in some hosts), but now likewise at rate e_0 this new symbiont is lost from hosts in the population (such that community S can revert to community s in some hosts). The resulting equations for the female hosts are then given by

$$\begin{aligned}\frac{\mathrm{d}x^+}{\mathrm{d}t} = \frac{b_0}{2}\Bigg(&\frac{\Gamma\left(V^{♀}+V^{♂}\right)\left(x^+z^+(\alpha(V^{♀})+\beta(V^{♂})-\alpha(V^{♀})\beta(V^{♂}))+\alpha(V^{♀})x^+z^-+\beta(V^{♂})x^-z^+\right)}{x^-+x^+}\\ &+\frac{\Gamma\left(V^{♀}+0\right)\left(x^+y^+(\alpha(V^{♀})+\beta(0)-\alpha(V^{♀})\beta(0))+\alpha(V^{♀})x^+y^-+\beta(0)x^-y^+\right)}{x^-+x^+}\Bigg)\\ &-\frac{x^+(d_0+d'\nu N)}{w}+e_0(x^--x^+) \end{aligned} \tag{5}$$

$$\begin{aligned}\frac{\mathrm{d}x^-}{\mathrm{d}t} = \frac{b_0}{2}\Bigg(&\frac{\Gamma\left(V^{♀}+V^{♂}\right)\left(x^-z^-+(1-\alpha(V^{♀}))x^+z^-+(1-\beta(V^{♂}))x^-z^++(1-\alpha(V^{♀}))(1-\beta(V^{♂}))x^+z^+\right)}{x^-+x^+}\\ &+\frac{\Gamma\left(V^{♀}+0\right)\left(x^-y^-+(1-\alpha(V^{♀}))x^+y^-+(1-\beta(0))x^-y^++(1-\alpha(V^{♀}))(1-\beta(0))x^+y^+\right)}{x^-+x^+}\Bigg)\\ &-x^-(d_0+d'\nu N)+e_0(x^+-x^-) \end{aligned} \tag{6}$$

where $N = x^+ + y^+ + z^+ + x^- + y^- + z^-$. Meanwhile for male uniparental hosts (carrying the M^- modifier gene that suppresses lactation in males) we have

$$\begin{aligned}\frac{\mathrm{d}y^+}{\mathrm{d}t} &= \frac{b_0}{2}\frac{\Gamma\left(V^{♀}+0\right)\left(x^+y^+(\alpha(V^{♀})+\beta(0)-\alpha(V^{♀})\beta(0))+\alpha(V^{♀})x^+y^-+\beta(0)x^-y^+)\right)}{x^-+x^+}\\ &\quad-\frac{y^+(d_0+d'\nu N)}{w}+e_0(y^--y^+) \end{aligned} \tag{7}$$

$$\begin{aligned}\frac{\mathrm{d}y^-}{\mathrm{d}t} &= \frac{b_0}{2}\frac{\Gamma\left(V^{♀}+0\right)\left(x^-y^-+(1-\alpha(V^{♀}))x^+y^-+(1-\beta(0))x^-y^++(1-\alpha(V^{♀}))(1-\beta(0))x^+y^+\right)}{x^-+x^+}\\ &\quad-y^-(d_0+d'\nu N)+e_0(y^+-y^-)\,. \end{aligned} \tag{8}$$

Finally, for male biparental hosts (carrying the M^+ modifier gene that allows for lactation in males) we have

$$\begin{aligned}\frac{\mathrm{d}z^+}{\mathrm{d}t} &= \frac{b_0}{2}\frac{\Gamma\left(V^{♀}+V^{♂}\right)\left(x^+z^+(\alpha(V^{♀})+\beta(V^{♂})-\alpha(V^{♀})\beta(V^{♂}))+\alpha(V^{♀})x^+z^-+\beta(V^{♂})x^-z^+\right)}{x^-+x^+}\\ &\quad-\frac{z^+(d_0+d'\nu N)}{w}+e_0(z^--z^+) \end{aligned} \tag{9}$$

$$\begin{aligned}\frac{\mathrm{d}z^-}{\mathrm{d}t} &= \frac{b_0}{2}\frac{\Gamma\left(V^{♀}+V^{♂}\right)\left(x^-z^-+(1-\alpha(V^{♀}))x^+z^-+(1-\beta(V^{♂}))x^-z^++(1-\alpha(V^{♀}))(1-\beta(V^{♂}))x^+z^+\right)}{x^-+x^+}\\ &\quad-z^-(d_0+d'\nu N))+e_0(z^+-z^-)\,. \end{aligned} \tag{10}$$

With Eqs. (5-10) in hand, we can now proceed to investigate the stability of a resident population of females (x^+ and x^- with $V^{♀} = 1$) and non-lactating males (y^+ and y^- with $V^{♂} = 0$) to invasion by lactating males (z^+ and z^- with $V^{♂} > 0$) that are introduced at low frequency. Varying both the relative fitness of hosts carrying the new symbiont (with community S), w, and the volume of milk produced by the invading male, $V^{♂}$, we observe a sharp transition between uniparental and biparental lactation, as illustrated in Figure 2.

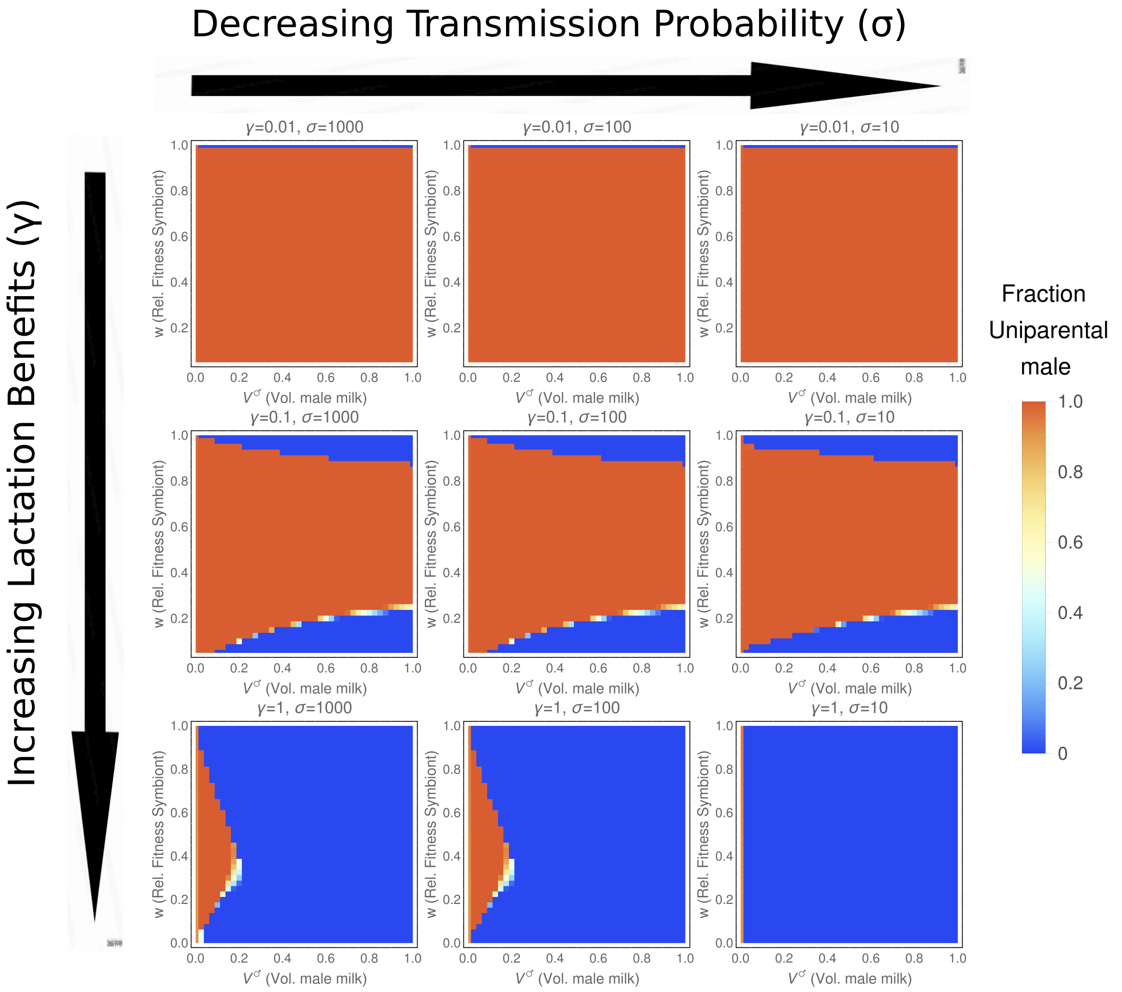

Supplementary Figure 2. Stability of a population of resident non-lactating males ($V^{♂} = 0$) to invasion by lactating males ($V^{♂} > 0$, see horizontal axis). Color indicates the fraction of uniparental non-lactating males, $(y^+ + y^-)/(y^+ + y^- + z^+ + z^-)$), after simulating Eqs. (5-10) between $t = 0$ and $t = 10^4$ starting from a low initial frequency of male lactation ($z^+(0) + z^-(0) = 2 \times 10^{-2}$). The resident population of non-lactating males population remains robust to invasion by males lactating with low milk volume, $V^{♂}$, so long as symbiont transmission can occur at low milk volume (σ is large) and the benefits of male lactation are moderate (γ is small). Remaining parameters are $b_0 = 10$, $d_0 = 1$, $d'\nu = 1$ and $e_0 = 0.6$. Simulations are conducted using NDSolve in Mathematica (see Main Text Code Availability Statement).

In Figure 2 we see that strictly maternal transmission of the microbiome (red regions) is selected for, and conversely male lactation selected against, whenever the benefits of male lactation are lower (smaller γ) and the transmission probability of the symbiote is high (larger σ). These results are intuitively clear. However perhaps more interestingly, we also see that strictly maternal transmission of the microbiome is generally robust to invasion from lactating males so long as the invading lactating males produce milk at low volume (small $V^{♂}$, see horizontal axis of plots in Figure 2).

B. Exploring the invasion potential of reduced male lactation in a resident lactating male population

Having shown that a resident population of non-lactating males is resistant to invasion from males that lactate at low volume, we now move on to consider the opposite scenario. Namely, we wish to determine if a resident population of lactating males is resistant or susceptible to invasion by males that lactate at a slightly lower volume to the residents.

We follow a similar approach to Section Supplementary Methods 1 A. Again we account for the density of female hosts, x^+ and x^- (with communities S and s respectively), who we assume to be capable of lactation at volume $V^{♀}$. However now we have that biparental lactating males (types z^+ and z^-) are resident and lactating at volume $V^{♂}$, while types y^+ and y^- are also biparental but lactating at a reduced volume $V^{♂}-\delta$. Note that setting $V^{♂}=1$ and $\delta=1$ then recovers the extreme case of a fully non-lactating male competing against a resident male lactating at full capacity. Eqs. (5-10) are then amended to read

$$\begin{aligned}
\frac{\mathrm{d}x^+}{\mathrm{d}t} &= \frac{b_0}{2}\left(\frac{\Gamma\left(V^{♀}+V^{♂}\right)\left(x^+z^+(\alpha(V^{♀})+\beta(V^{♂})-\alpha(V^{♀})\beta(V^{♂}))+\alpha(V^{♀})x^+z^-+\beta(V^{♂})x^-z^+\right)}{x^-+x^+}\right.\\
&\left.+\frac{\Gamma\left(V^{♀}+V^{♂}-\delta\right)\left(x^+y^+(\alpha(V^{♀})+\beta(V^{♂}-\delta)-\alpha(V^{♀})\beta(V^{♂}-\delta))+\alpha(V^{♀})x^+y^-+\beta(V^{♂}-\delta)x^-y^+\right)}{x^-+x^+}\right)\\
&-\frac{x^+(d_0+d'\nu N)}{w}+e_0(x^--x^+) \qquad (11)\\
\frac{\mathrm{d}x^-}{\mathrm{d}t} &= \frac{b_0}{2}\left(\frac{\Gamma\left(V^{♀}+V^{♂}\right)\left(x^-z^-+(1-\alpha(V^{♀}))x^+z^-+(1-\beta(V^{♂}))x^-z^++(1-\alpha(V^{♀}))(1-\beta(V^{♂}))x^+z^+\right)}{x^-+x^+}\right.\\
&\left.+\frac{\Gamma\left(V^{♀}+V^{♂}-\delta\right)\left(x^-y^-+(1-\alpha(V^{♀}))x^+y^-+(1-\beta(V^{♂}-\delta))x^-y^++(1-\alpha(V^{♀}))(1-\beta(V^{♂}-\delta))x^+y^+\right)}{x^-+x^+}\right)\\
&-x^-(d_0+d'\nu N)+e_0(x^+-x^-) \qquad (12)
\end{aligned}$$

$$\begin{aligned}
\frac{\mathrm{d}y^+}{\mathrm{d}t} &= \frac{b_0}{2}\frac{\Gamma\left(V^{♀}+V^{♂}-\delta\right)\left(x^+y^+(\alpha(V^{♀})+\beta(V^{♂}-\delta)-\alpha(V^{♀})\beta(V^{♂}-\delta))+\alpha(V^{♀})x^+y^-+\beta(V^{♂}-\delta)x^-y^+)\right)}{x^-+x^+}\\
&-\frac{y^+(d_0+d'\nu N)}{w}+e_0(y^--y^+) \qquad (13)\\
\frac{\mathrm{d}y^-}{\mathrm{d}t} &= \frac{b_0}{2}\frac{\Gamma\left(V^{♀}+V^{♂}-\delta\right)\left(x^-y^-+(1-\alpha(V^{♀}))x^+y^-+(1-\beta(V^{♂}-\delta))x^-y^++(1-\alpha(V^{♀}))(1-\beta(V^{♂}-\delta))x^+y^+\right)}{x^-+x^+}\\
&-y^-(d_0+d'\nu N)+e_0(y^+-y^-)\,. \qquad (14)
\end{aligned}$$

$$\begin{aligned}
\frac{\mathrm{d}z^+}{\mathrm{d}t} &= \frac{b_0}{2}\frac{\Gamma\left(V^{♀}+V^{♂}\right)\left(x^+z^+(\alpha(V^{♀})+\beta(V^{♂})-\alpha(V^{♀})\beta(V^{♂}))+\alpha(V^{♀})x^+z^-+\beta(V^{♂})x^-z^+\right)}{x^-+x^+}\\
&-\frac{z^+(d_0+d'\nu N)}{w}+e_0(z^--z^+) \qquad (15)\\
\frac{\mathrm{d}z^-}{\mathrm{d}t} &= \frac{b_0}{2}\frac{\Gamma\left(V^{♀}+V^{♂}\right)\left(x^-z^-+(1-\alpha(V^{♀}))x^+z^-+(1-\beta(V^{♂}))x^-z^++(1-\alpha(V^{♀}))(1-\beta(V^{♂}))x^+z^+\right)}{x^-+x^+}\\
&-z^-(d_0+d'\nu N))+e_0(z^+-z^-)\,. \qquad (16)
\end{aligned}$$

With Eqs. (11-16) in hand, we can now proceed to investigate the stability of a resident population of females (x^+ and x^- with $V^{♀}=1$) and males lactating at maximum capacity (z^+ and z^- with $V^{♂}=1$) to invasion by males that lactate at reduced volume (y^+ and y^- with $V^{♂}=1-\delta$) that are introduced at low frequency. Varying both the relative fitness of hosts carrying the new symbiont (with community S), w, and the reduction in the volume of milk produced by the invading male, δ, we again observe a sharp transition between uniparental and biparental lactation, as illustrated in Figure 3.

In Figure 3 we see that male lactation at high volume (blue regions) is selected for, and conversely male lactation at lower volumes selected against, whenever the benefits of male lactation are moderate

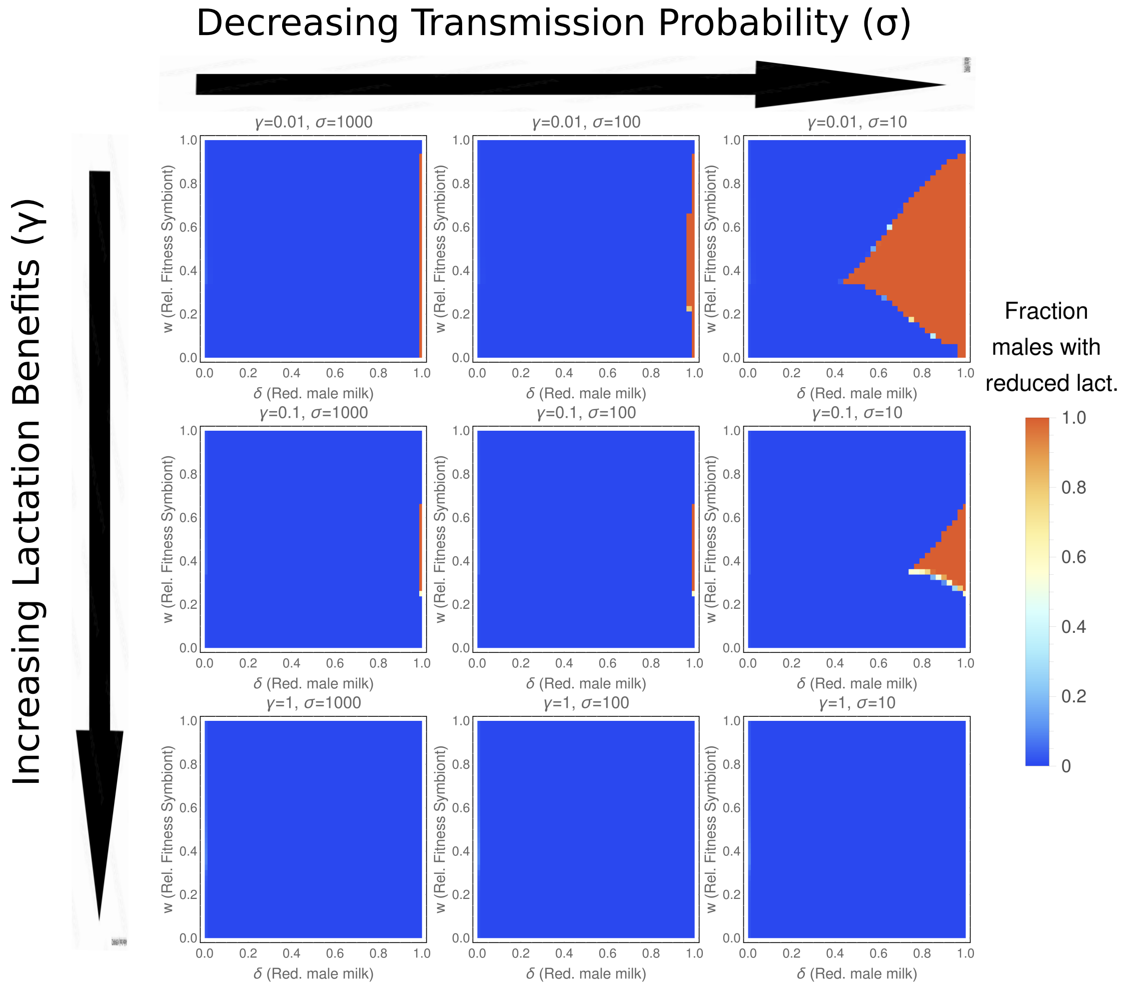

Supplementary Figure 3. Susceptibility of a population of resident lactating males ($V^{\male} = 1$) to invasion by males lactating at a reduced volume ($V^{\male} = 1 - \delta$, see horizontal axis). Color indicates the fraction of males lactating at a reduced volume, $(y^+ + y^-)/(y^+ + y^- + z^+ + z^-)$), after simulating Eqs. (11-16) between $t = 0$ and $t = 10^4$ starting from a low initial frequency of male lactation ($z^+(0) + z^-(0) = 2 \times 10^{-2}$). The resident population of males lactating at high volume ($V^{\male} = 1$) remains robust to invasion by males lactating with lower milk volume ($V^{\male} = 1 - \delta$), so long as the reduction in milk volume δ is small. Remaining parameters are $b_0 = 10$, $d_0 = 1$, $d'\nu = 1$ and $e_0 = 0.6$. Simulations are conducted using NDSolve in Mathematica (see Main Text Code Availability Statement).

to high (larger γ). This result is intuitively clear. The success of males lactating with a reduced milk volume is now dependent on a low transmission probability of the microbiome (small σ). This is because the form of the transmission function, $\beta(V^{\male})$, means that males reducing their lactation volume from $V^{\male}$ to $V^{\male} - \delta$ do little to reduce the risk of transmitting a deleterious symbiote to their offspring while still paying a high cost to their offspring survival probability (see Figure 1). Most importantly, we also see that male lactation at high volume is robust to invasion from males lactating at lower volumes so long as the reduction in milk volume is low (small δ, see horizontal axis of plots in Figure 3).
