# Supplementary material for: Maternal transmission as a microbial symbiont sieve, and the absence of lactation in male mammals
Source: Nat Commun. 2024 Jun 27;15:5341. doi: 10.1038/s41467-024-49559-5 (PMC11211401; doi:10.1038/s41467-024-49559-5)
Supplement: Supplementary file 3 — Description of Additional Supplementary Files [file 41467_2024_49559_MOESM3_ESM.pdf]

File Name: Supplementary Data 1
Description: Contains all data used by gnuplot in the figures in the main text.

File Name: Supplementary Code 1
Description: Contains all code used to produce the data (written in C), to conduct analyses (written in Mathematica), or to plot the data (written using gnuplot).
