# Supplementary material for: Maternal transmission as a microbial symbiont sieve, and the absence of lactation in male mammals
Source: Nat Commun. 2024 Jun 27;15:5341. doi: 10.1038/s41467-024-49559-5 (PMC11211401; doi:10.1038/s41467-024-49559-5)
Supplement: Supplementary file 5 — Supplementary Code 1 [file 41467_2024_49559_MOESM5_ESM.zip › Supplementary Code 1/readme.pdf]

Maternal transmission as a microbial symbiont sieve, and the absence of lactation in male mammals

Brennen T. Fagan*
Leverhulme Centre for Anthropocene Biodiversity, University of York and
Department of Mathematics, University of York

George W. A. Constable and Richard Law
Department of Mathematics, University of York
(Dated: June 6, 2024)

CONTENTS

Introduction 1
I. File Name: C_code.c 2
II. File Name: C_code_20spp.c 3
III. File Name: Mathematica-DynSys.nb 4
IV. File Name: Mathematica-Figure5.nb 4
V. File Name: Maternal_transmission_SI_code.nb 5
VI. Minor Files 5
VII. Folder: figure_2 6
VIII. Folder: figure_3 7
IX. Folder: figure_4 9
X. Folder: figure_5 10

INTRODUCTION

In this file, we describe first supplementary code accompanying "Maternal transmission as a symbiont sieve, and the absence of lactation in male mammals", by Brennen Fagan, George W. A. Constable, and Richard Law. We then describe supplementary data files.

Five programs/notebooks are included:

- C_code.c, by Richard Law
- C_code_20spp.c, By Richard Law
- Mathematica-DynSys.nb, by Brennen T. Fagan
- Mathematica-Figure5.nb, by Brennen T. Fagan
- Maternal_transmission_SI_code.nb, by George W. A. Constable

Each .c file section is ended with a description of parameters used to recreate the images in the main text. Each .nb file's default parameters should yield the relevant plots, where are generally stored in the notebook until the file is re-run.

Mathematica code by Brennen T. Fagan was last edited using Mathematica 12.1.1.0. We adopt the convention therein of referring to the populations by their (mean-field) densities. Variable names are generally the same as in the main text except as follows:

- $x^+ \to x$
- $x^- \to y$
- $e_0 \to m$

In order to reproduce the figures generated from data, we include four folders in the Supplementary Code and Supplementary Data each, labelled figure_#. (Figure 1 is a schematic assembled in OpenOffice Draw with no corresponding data.) The supplementary code includes the gnuplot code used to generate the figure, as well as subfolders that contain the scripts to produce the data in the (matching) subfolder in the Supplementary Data folders. Note that the gnuplot scripts are set to produce postscript files (.ps). Additionally, OpenOffice Draw was used to organise Figure 2.

The supplementary data files/folders provided are:

figure_2/ 13_iterate_d_div_Wsym_sd_0.30_for_paper, which contains 4 folders containing the elements of the bar charts in Figure 2 and line1.dat (which defines a vertical line at the point of neutrality).

14_timeser_d_div_Wsym_sd_0.30_for_paper, which contains 2 folders each containing 1 time series, one for biparental and one for maternal transmission.

figure_3/ which contains 2 folders (one for each transmission type) and 6 files defining lines. The folders contain the time series and the microbiome frequency bar chart data.

figure_4/ 01_symstart_0.05_w_0.6, which contains one time series file.

figure_5/ 01_biparental, which contains 2 folders corresponding to a time series realisation and a map of the regions.

02_maternal/map, which corresponds to a map of the regions.

03_biparental_ODE, which contains a time series of the mean-field ODE dynamics.

I. FILE NAME: C_CODE.C

C_code.c is the primary engine for creating the data and experiments described in the main text. After making any required modifications (e.g. changing parameter values), you will need to compile C_code.c and run the resulting output file using a standard C compiler (e.g. we use C11 with compiler GCC 7.5.0 included with Ubuntu 18.04.6 LTS). Note that demographic stochasticity will in theory lead to different results with each realisation, which is why we provide random seeds in some locations (i.e. to retain consistency with the specific results plotted in the paper).

In detail, we keep constants controlling the behaviour of the simulations generally at the top (lines 21 – 57), with descriptions therein. (Note that this file and C_code_20spp.c adopt a consistent 1-indexed standard as opposed to the default 0-indexing.) We also define some simple types in order to keep data bundled together (lines 64 – 121), as well as some standard global variables (lines 128 – 140). Note that lines 143 – 150 provide flags for more verbose output. We also provide a standard helper function for generating normally distributed samples (lines 156 – 170, e.g. equation 7.2.10 on page 289 of Numerical Recipes in C Second Edition by Press, Teukolsky, Vetterling, and Flannery). Implementation of the initial conditions and species parameters (as well as the initial print of the detailed data) can be found starting on line 179. The standardised output function can be found starting on line 249 (which navigates the data by linked list).

Line 282 begins the construction of the linked list governing the population, randomly assigning each initialized individual a sex, setting birth rate (0 if male), setting death rate, setting modifier gene for males determining transmission type, and finally randomly allocating the symbionts (and setting associated benefit/penalty of the novel symbiont). Line 433 starts the function which collects the probabilities for individual events, which are then linearly processed and used by choose_event on line 489 in a standard Gillespie algorithm (citation 64 in the main text). The choose_dad function on line 625 behaves similarly. The symbiont_transmission function, line 682, implements the vertical transmission from parent(s) to a newborn host using simple switches.

The birth function, starting line 760, manages the linked list, allocating the new memory, using choose_dad and symbiont_transmission to determine the newborn's properties including modifier gene if appropriate, randomly assigning sex, and then assigning event propensities as above. The simpler death function starts on line 912 and the horizontal transmission function begins on line 963. In order to construct the time series data files (output.timeseries.dat), a simple scan and record scheme is begins on line 990. For a single realisation, we provide the wrapper function simulate on 1099, which calls the initialisation steps, manages the time loop, calls the event steps, and calls the time series recording function. Finally, the scan for the fitness frequency distribution is implemented in fitness_freq on line 1170, before the main function (which performs file management) occurs on line 1202. Note that random selection of the symbiont's properties occurs on lines 1264 - 1270.

To reproduce the results in the figures, the code needs to be compiled with the settings below.

Figure 2b

This uses the default settings of the code.

Output: frequency distribution of wsym in output.freq.dist.dat

Figure 2c

Line 30: set method_vertical = 0

Output: frequency distribution of wsym in output.freq.dist.dat

Figure 2d,e

Line 30: for biparental transmission, set method_vertical = 2
for maternal transmission, set method_vertical = 0
Line 42: set tmax = 10
Line 53: set niterate = 1
Line 150: set flag_timeseries = 1
Line 1265: to 1267: comment out these lines
Line 1269: remove comment brackets and set wsym to values in the figure
Output: time series in output.timeseries.dat

Figure 4

Line 30: set method_vertical = 3
Line 42: set tmax = 20
Line 44: set Nsymstart = 100
Line 46: set fmodstart = 0.2
Line 53: set niterate = 1
Line 150: set flag_timeseries = 1
Line 1265 – 1267: comment out these lines
Line 1269: remove comment brackets and set wsym = 0.6
Seed: 725
Output: time series in output.timeseries.dat
Note: More than one realization may be needed to get an instance of complete fixation of the the modifier gene M^-

Figure 5c

Line 30: set method_vertical = 3
Line 36: set method_horizontal = 1
Line 42: set tmax = 100
Line 43: set Nstart = 656
Line 44: set Nsymstart = 80
Line 46: set fmodstart = 0.02
Line 53: set niterate = 1
Line 150: set flag_timeseries = 1
Line 1265 to 1267: comment out these lines
Line 1269: remove comment brackets and set wsym = 0.37
Seed: 470
Output: time series in output.timeseries.dat
Note: More than one realization may be needed to get an instance of complete fixation of the the modifier gene M^-

II. FILE NAME: C_CODE_20SPP.C

C_code_20spp.c is a modification of C_code.c that expands the effective number of taxa in the microbiome. The taxa are again assumed to be independent of one another, so that the host fitness is 1 if there are no symbionts and the average of the symbiont induced fitnesses if any symbionts are present.

In detail, there are a few differences between C_code_20spp.c and C_code.c. First, there is the obvious expanded number of taxa in the microbiome (line 53, alongside storage in rep, beginning line 83). With multiple taxa, we

also need to define how they interact, here by averaging (see line 32 and lines 315 – 338). This naturally requires modifying the initialization as well which begins much the same (line 346). Changes are in the order of evaluation and incorporating the effects of the more complex microbiome on the host, which requires scanning and then evaluating the microbiome in addition to the usual uniformly at random allocation of symbionts. Note that effects on host intrinsic birth rate or solely host intrinsic death rate are disabled in this version (method_sym set to 1, 2, or 3). Transmission of the microbiome to a newborn (beginning line 796) naturally must include scanning over the parents' entire microbiomes (with conditionality on the father's transmission strategy). Note that horizontal transmission of multiple symbionts is not implemented (line 1103). Additionally, there are two versions of the time series formatting function (lines 1131 and 1233); the multi-taxon version does not report the coefficient of disequilibrium, but reports every taxon's progress. The main function then includes some additional checks to make sure that incompatible combinations of parameters are not used.

Figure 3

Line 41: for biparental transmission, set method_vertical = 2
for maternal transmission, set method_vertical = 0
Seed: 7597

III. FILE NAME: MATHEMATICA-DYNSYS.NB

The file Mathematica-DynSys.nb is the tool we used to analyse and robustness check the system. It comprises an implementation of the dynamical system as a manipulate object, which allows the user to dynamically change the parameters used. Note that there are three methods to dynamically change the parameters of the system. Initial conditions can be specified by clicking on the phase portrait. Individual parameters can be changed by moving the sliders as well as by pressing the "+" button on the right of a slider for more precise manipulation. Additionally, we display all detected fixed points above the phase portrait.

In detail, the outer manipulate block specifies the object to be manipulated as its first argument and the parameters to manipulate the object over as the second argument. The latter includes

$b0$, per-female probability per unit time of giving birth,

$d0$, intrinsic death rate,

$dprime$, the scaling on the density-dependent component of death,

v, our scaling parameter,

α, the probability that a female passes on the novel symbiont,

β, the probability that a male passes on the novel symbiont,

w, the fitness of hosts carrying the novel symbiont (relative to those not),

m, the (linear) environmental horizontal transmission,

T, the time over which the dynamical system is numerically integrated, and

$point$, the initial conditions in density space of the dynamical system.

The remainder of the code defines the model as *system*, solves the fixed points as $fixpts$, and then uses stream plots to create a phase portrait over which a single solution and its trajectory are plotted (using parametric plots and the numeric differential equation solver).

IV. FILE NAME: MATHEMATICA-FIGURE5.NB

The file Mathematica-Figure5.nb is the tool we used to categorise fixed points as a function of the parameters. Helper functions are defined at the top of the file. The main function is below and recreates a Mathematica version of Figure 5 in the main text. The Mathematica version can be manipulated by re-running the module with different parameter values. The resultant chart will report the birth and death parameters used to construct it (above), as well as all fixed point combinations identified (by color, below). Note also that the chart is plotted "pixel-by-pixel";

zooming in will separate out the pixels. The number of pixels plotted can be increased by changing the step sizes of m and w (in the module declaration).

In detail, our helper functions

- **limitIfSubFails** which replaces direct numerical evaluation with a limit evaluation.
- **JacobianMatrix** and JacobianDeterminant, imported from `https://mathworld.wolfram.com/Jacobian.html`.
- **valIfNotComplexAndPhysicalAndStable** which filters out points that are not-physical or are not stable.
- **categorizeIDsNoXY** which creates the coding scheme used in our plots.
- **categorizeNoXY** which applies the coding scheme to a list of points.
- **makeChart** which wraps the entire procedure together by gathering the rules, identifying the fixed points, evaluating and categorizing the fixed points, and then configuring them for plotting as a list.

The main function then reduces to supplying parameters to a Module, setting the system as in Mathematica-DynSys.nb with the same variables (note the slight change in notation to bv and dv from $b0$ and $d0$), and running the wrapper function. Note the time and memory taken depend heavily on the step sizes chosen for the axes.

V. FILE NAME: MATERNAL_TRANSMISSION_SI_CODE.NB

The file Maternal_transmission_SI_code.nb contains the code that we used to generate the figures in our supplementary information. This supplementary information examines the implications of the benefits of lactation on the population by separately considering the benefits as offspring survival probability and risks as symbiont transmission probability. In the first section, a population with uniparental lactation is subject to invasion by a small biparental invasive population. In the second section, these roles are reversed. In both sections, the benefits and risks are varied between subsections and the volume of milk of the invasive population and danger of an environmentally acquired symbiont (that is nonetheless transmissable via lactation) are plotted against the resulting fraction of males that are uniparental or experience reduced lactation.

In detail, this file is written in a full notebook format, starting with the biparental invasion of a uniparental population. First we reproduce the Supplementary Figure 1. We then construct each panel inside Supplementary Figure 2 inside its own subsection. Each panel requires evaluating a grid of points throughout the parameter space defined by milk volumes and fitness of the symbiont. The system is redefined for each point, fixed points are solved for to verify there is only one in the system, and then the system is numerically integrated. The resulting ratio of uniparental to biparental males is stored and then plotted. The same procedure is then performed for the different initial conditions in Supplementary Figure 3.

VI. MINOR FILES

A. File Name: gaussian.c

This file is located in figure_2/13_iterate_d_div_Wsym_sd_0.30_for_paper/03_gaussian. This file evaluates a (truncated) normal distribution (mean of 1, standard deviation of 0.3) at 0.05 steps from 0 to 2.2, upscaled by the number of draws (5,000) used in Figure 2. Both the value at the point and the sum up to that point are evaluated.

B. File Name: dynamics.c

This file is located in figure_5/03_biparental_ODE. This file numerically integrates the equations listed in the main text at the end of the section Methods: Differential equation model. It begins by listing out the parameters (with commented descriptions alongside the parameters). Note the usage of beta_M0 and beta_M1, which control the implementation of the transmission types used by males. It then defines the format of output.timeseries.dat in the output function (line 91). The functions birth_rates (line 128), death_rates (line 192), and encounter_rates (line 211) calculate the birth, death, and (environmental) horizontal transmission rates as functions of the system parameters.

The system's initial densities are then initialised near to the frequencies of the corresponding stochastic simulation in figure_5/simulate_w_0.37_E0_0.1/output.timeseries.dat, see lines 259 – 272. The main integration loop then occurs (line 279) by reporting the current results, calculating the rates, multiplying them by the step sizes, and adjusting the population and time.

C. Gnuplot files

Corresponding to each panel in our figures, we provide (standard) gnuplot code, which describes the images in each panel. Each gnuplot file can produce multiple panels and can source data from the appropriate data files if moved from the Supplementary Code directory to the corresponding Supplementary Data directory. (E.g. gnu.fig5.horizontal in Supplementary Code 1/figure_5/ would be moved to Supplementary Data/figure_5/.)

VII. FOLDER: FIGURE_2

A. Folder: 13_iterate_d_div_Wsym_sd_0.30_for_paper

1. File Name: 00_symbiont_fitness_distribution/output.freq.distrib.dat

This file contains 3 columns: histogram bar starting locations, histogram bar (visual) ending locations, and the count of the number of random draws from the truncated normal distribution that fell into the corresponding bin. It was created by simulate.c in the parallel Supplementary Code directory, which is a derivative of C_code.c.

2. File Name: 01_biparental_transmission/output.freq.distrib.dat

This file contains 3 columns: histogram bar starting locations, histogram bar (visual) ending locations, and the count of the number of simulations that had at least 10% of individuals with the symbiont at the end of a simulation. It was created by simulate.c in the parallel Supplementary Code directory, which is a derivative of C_code.c.

3. File Name: 02_maternal_transmission/output.freq.distrib.dat

This file contains 3 columns: histogram bar starting locations, histogram bar (visual) ending locations, and the count of the number of simulations that had at least 10% of individuals with the symbiont at the end of a simulation. It was created by simulate.c in the parallel Supplementary Code directory, which is a derivative of C_code.c.

4. File Name: 03_gaussian/output.gaussian.dat

This file contains 3 columns: x axis locations, the plotted y axis values for the Gaussian in Figure 2 (adjusted for the number of draws from the distribution of fitness w of hosts carrying the new symbiont), and summed y axis values. It was created by gaussian.c.

5. File Name: line1.dat

This file contains 2 columns defining the starting and ending points of the vertical line in Figure 2.

B. Folder: 14_timeser_d_div_Wsym_sd_0.30_for_paper

1. File Name: 01_biparental_transmission/output.timeseries.Wsym_0.6.dat

This file contains whitespace separated columns with a header designated by the # character. The headings correspond to 'time', the species reported 'sp', the index of the event reported 'nevent', the type of 'event' ('d' for

death, 'b' for birth), the current population size 'n' with a breakdown of females and males 'nf' and 'nm'. The fraction of the population with the symbiont 'fsymbiont' is then followed by the number of males with the maternal and biparental transmission 'nmat' and 'nbip'. The fraction of males with maternal transmission is 'fmaternal'. The combination of transmission state 'mat' (maternal) or 'bip' (biparental) and symbiont state amongst males negative '-' or positive '+' follows before the coefficient of disequilibrium 'diseq' (see Methods: Stochastic birth-death model). This file was created by simulate.c in the parallel Supplementary Code directory, which is a derivative of C_code.c.

2. File Name:02_maternal_transmission/output.timeseries.Wsym_0.6.dat

This file contains whitespace separated columns with a header designated by the # character. The headings correspond to 'time', the species reported 'sp', the index of the event reported 'nevent', the type of 'event' ('d' for death, 'b' for birth), the current population size 'n' with a breakdown of females and males 'nf' and 'nm'. The fraction of the population with the symbiont 'fsymbiont' is then followed by the number of males with the maternal and biparental transmission 'nmat' and 'nbip'. The fraction of males with maternal transmission is 'fmaternal'. The combination of transmission state 'mat' (maternal) or 'bip' (biparental) and symbiont state amongst males negative '-' or positive '+' follows before the coefficient of disequilibrium 'diseq' (see Methods: Stochastic birth-death model). This file was created by simulate.c in the parallel Supplementary Code directory, which is a derivative of C_code.c.

VIII. FOLDER: FIGURE_3

A. Folder: 01_biparental_transmission

1. File Name: output.freq.microbiome.size.t_0.0.dat

This file contains whitespace separated columns with a header designated by the # character and is used to generate a histogram seen in Figure 3. The 't' in the file's title denotes the time point that the population's state was measured at. The first column designates the number of symbiont taxa hosted by an individual 'taxa'. The second column 'Frequency(n)' denotes the number of individuals with the corresponding number of taxa. The third column 'propn' normalises 'Frequency(n)'.

Beneath that is a record of each individual as well as the presence-absence bitstring of symbionts and the number of taxa present for that individual. This was not used in the present study.

This file was created by simulate.c in the parallel Supplementary Code directory, which is a derivative of C_code.c.

2. File Name: output.freq.microbiome.size.t_2.0.dat

This file contains whitespace separated columns with a header designated by the # character and is used to generate a histogram seen in Figure 3. The 't' in the file's title denotes the time point that the population's state was measured at. The first column designates the number of symbiont taxa hosted by an individual 'taxa'. The second column 'Frequency(n)' denotes the number of individuals with the corresponding number of taxa. The third column 'propn' normalises 'Frequency(n)'.

Beneath that is a record of each individual as well as the presence-absence bitstring of symbionts and the number of taxa present for that individual. This was not used in the present study.

This file was created by simulate.c in the parallel Supplementary Code directory, which is a derivative of C_code.c.

3. File Name: output.freq.microbiome.size.t_4.0.dat

This file contains whitespace separated columns with a header designated by the # character and is used to generate a histogram seen in Figure 3. The 't' in the file's title denotes the time point that the population's state was measured at. The first column designates the number of symbiont taxa hosted by an individual 'taxa'. The second column 'Frequency(n)' denotes the number of individuals with the corresponding number of taxa. The third column 'propn' normalises 'Frequency(n)'.

Beneath that is a record of each individual as well as the presence-absence bitstring of symbionts and the number of taxa present for that individual. This was not used in the present study.

This file was created by simulate.c in the parallel Supplementary Code directory, which is a derivative of C_code.c.

4. File Name: output.timeseries.dat

This file contains whitespace separated columns with a header designated by the # character and is used to generate a time series seen in Figure 3. The header is two lines long. On the first line, 'time' designates the simulation time at which the row is generated. The column 'sp' denotes the host species. The index of the event reported is column 'nevent' and the type of event 'event' ('d' for death, 'b' for birth). The current population size is represented by 'n'. The (first order) fitness of a host with a given symbiont is then captured by the 'w1' values, of which there are 20, one for each symbiont. Beneath that in the second line is the unused second order fitness effect. Each (first and second order) fitness effect column is used to record the frequency of the corresponding symbiont in the population. Analogous columns (without header) follow and capture the counts of individuals with the corresponding symbiont in the population. This file was created by simulate.c in the parallel Supplementary Code directory, which is a derivative of C_code.c. Please see Figure 3 and the Supplementary Code for implementation details.

B. Folder: 02_maternal_transmission

1. File Name: output.freq.microbiome.size.t_0.dat

This file contains whitespace separated columns with a header designated by the # character and is used to generate a histogram seen in Figure 3. The 't' in the file's title denotes the time point that the population's state was measured at. The first column designates the number of symbiont taxa hosted by an individual 'taxa'. The second column 'Frequency(n)' denotes the number of individuals with the corresponding number of taxa. The third column 'propn' normalises 'Frequency(n)'.

Beneath that is a record of each individual as well as the presence-absence bitstring of symbionts and the number of taxa present for that individual. This was not used in the present study.

This file was created by simulate.c in the parallel Supplementary Code directory, which is a derivative of C_code.c.

2. File Name: output.freq.microbiome.size.t_7.dat

This file contains whitespace separated columns with a header designated by the # character and is used to generate a histogram seen in Figure 3. The 't' in the file's title denotes the time point that the population's state was measured at. The first column designates the number of symbiont taxa hosted by an individual 'taxa'. The second column 'Frequency(n)' denotes the number of individuals with the corresponding number of taxa. The third column 'propn' normalises 'Frequency(n)'.

Beneath that is a record of each individual as well as the presence-absence bitstring of symbionts and the number of taxa present for that individual. This was not used in the present study.

This file was created by simulate.c in the parallel Supplementary Code directory, which is a derivative of C_code.c.

3. File Name: output.freq.microbiome.size.t_15.dat

This file contains whitespace separated columns with a header designated by the # character and is used to generate a histogram seen in Figure 3. The 't' in the file's title denotes the time point that the population's state was measured at. The first column designates the number of symbiont taxa hosted by an individual 'taxa'. The second column 'Frequency(n)' denotes the number of individuals with the corresponding number of taxa. The third column 'propn' normalises 'Frequency(n)'.

Beneath that is a record of each individual as well as the presence-absence bitstring of symbionts and the number of taxa present for that individual. This was not used in the present study.

This file was created by simulate.c in the parallel Supplementary Code directory, which is a derivative of C_code.c.

4. File Name: output.timeseries.dat

This file contains whitespace separated columns with a header designated by the # character and is used to generate a time series seen in Figure 3. The header is two lines long. On the first line, 'time' designates the simulation time at which the row is generated. The column 'sp' denotes the host species. The index of the event reported is column

'nevent' and the type of event 'event' ('d' for death, 'b' for birth). The current population size is represented by 'n'. The (first order) fitness of a host with a given symbiont is then captured by the 'w1' values, of which there are 20, one for each symbiont. Beneath that in the second line is the unused second order fitness effect. Each (first and second order) fitness effect column is used to record the frequency of the corresponding symbiont in the population. Analogous columns (without header) follow and capture the counts of individuals with the corresponding symbiont in the population. This file was created by simulate.c in the parallel Supplementary Code directory, which is a derivative of C_code.c. Please see Figure 3 and the Supplementary Code for implementation details.

C. File Name: line_t_0_bip

This file defines a line at time 0 for the biparental transmission plot.

D. File Name: line_t_0_mat

This file defines a line at time 0 for the maternal transmission plot.

E. File Name: line_t_2

This file defines a line at time 2 for the biparental transmission plot.

F. File Name: line_t_4

This file defines a line at time 4 for the biparental transmission plot.

G. File Name: line_t_7

This file defines a line at time 7 for the maternal transmission plot.

H. File Name: line_t_15

This file defines a line at time 15 for the maternal transmission plot.

IX. FOLDER: FIGURE_4

A. File Name: 01_symstart_0.05_w_0.6/output.timeseries.dat

This file contains whitespace separated columns with a header designated by the # character. The headings correspond to 'time', the species reported 'sp', the index of the event reported 'nevent', the type of 'event' ('d' for death, 'b' for birth), the current population size 'n' with a breakdown of females and males 'nf' and 'nm'. The fraction of the population with the symbiont 'fsymbiont' is then followed by the number of males with the maternal and biparental transmission 'nmat' and 'nbip'. The fraction of males with maternal transmission is 'fmaternal'. The combination of transmission state 'mat' (maternal) or 'bip' (biparental) and symbiont state amongst males negative '-' or positive '+' follows before the coefficient of disequilibrium 'diseq' (see Methods: Stochastic birth-death model). This file was created by simulate.c in the parallel Supplementary Code directory, which is a derivative of C_code.c.

X. FOLDER: FIGURE_5

A. Folder: 01_biparental

1. *File Name: map/lineAB.dat*

In Figure 5 panel a, this file defines the line in (w, e_0) space between "host bimorphic" and "host extinction". This file contains whitespace separated columns with a header designated by the #. Here e0 refers to the e_0 axis of horizontal transmission of the novel symbiont into the population. The w column refers to the w axis corresponding to the fitness of hosts with the novel symbiont.

This data corresponds to the lines produced by Mathematica-Figure5.nb. Advanced users will note that this is an intermediate output of the makeChart helper function and can be retrieved by returning "fixptTypes" instead of the visualisation; the precision desired can be customised using the step sizes "stepM" and "stepW" in that file.

2. *File Name: map/lineAC.dat*

In Figure 5 panel a, this file defines the line in (w, e_0) space between "host extinction" and "fixation of symbiont".

This data corresponds to the lines produced by Mathematica-Figure5.nb. Advanced users will note that this is an intermediate output of the makeChart helper function and can be retrieved by returning "fixptTypes" instead of the visualisation; the precision desired can be customised using the step sizes "stepM" and "stepW" in that file.

3. *File Name: map/point.dat*

In Figure 5 panels a and b, this file defines the triangular point at which the time series in panel c is evaluated.

4. *File Name: simulate_w_0.37_E0_0.1/output.timeseries.dat*

This file contains whitespace separated columns with a header designated by the # character. The headings correspond to 'time', the species reported 'sp', the index of the event reported 'nevent', the type of 'event' ('d' for death, 'b' for birth, 'e' for horizontal transmission), the current population size 'n' with a breakdown of females and males 'nf' and 'nm'. The fraction of the population with the symbiont 'fsymbiont' is then followed by the number of males with the maternal and biparental transmission 'nmat' and 'nbip'. The fraction of males with maternal transmission is 'fmaternal'. The combination of transmission state 'mat' (maternal) or 'bip' (biparental) and symbiont state amongst males negative '-' or positive '+' follows before the coefficient of disequilibrium 'diseq' (see Methods: Stochastic birth-death model). This file was created by simulate.c in the parallel Supplementary Code directory, which is a derivative of C_code.c.

B. Folder: 02_maternal/map

1. *File Name: map/lineAB.dat*

In Figure 5 panel b, this file defines the line in (w, e_0) space between "host bimorphic" and "host extinction".

This data corresponds to the lines produced by Mathematica-Figure5.nb. Advanced users will note that this is an intermediate output of the makeChart helper function and can be retrieved by returning "fixptTypes" instead of the visualisation; the precision desired can be customised using the step sizes "stepM" and "stepW" in that file.

2. *File Name: map/lineAC.dat*

In Figure 5 panel b, this file defines the line in (w, e_0) space between "host extinction" and "fixation of symbiont".

This data corresponds to the lines produced by Mathematica-Figure5.nb. Advanced users will note that this is an intermediate output of the makeChart helper function and can be retrieved by returning "fixptTypes" instead of the visualisation; the precision desired can be customised using the step sizes "stepM" and "stepW" in that file.

3. File Name: map/lineBC.dat

In Figure 5 panel b, this file defines the line in (w, e_0) space between “host bimorphic” and “fixation of symbiont”. This data corresponds to the lines produced by Mathematica-Figure5.nb. Advanced users will note that this is an intermediate output of the makeChart helper function and can be retrieved by returning “fixptTypes” instead of the visualisation; the precision desired can be customised using the step sizes “stepM” and “stepW” in that file.

C. File Name: 03_biparental_ODE/output.timeseries.dat

This file contains whitespace separated columns with a header row and is a result of numerically integrating the equations at the end of the Methods: Differential equation model. The heading ‘t’ corresponds to time. The heading ‘VX’ corresponds to the system volume multiplied by the sum of the population densities. The heading ‘fr_sym1’ corresponds to the frequency of the novel symbiont in the population, while ‘fr_M0’ tracks the frequency of the males that rely on maternal transmission out of the entire population of males. The heading ‘D’ is the coefficient of disequilibrium, after which follows the effective population sizes of each subpopulation. In order, these are the volume multiplied by the density of females without the symbiont ‘x.fsym0’, females with the symbiont ‘x.fsym1’, uniparental males without the symbiont ‘x.msym0M0’, uniparental males with the symbiont ‘x.msym1M0’, biparental males without the symbiont ‘x.msym0M1’, and biparental males with the symbiont ‘x.msym1M1’. This file was created by dynamics.c in the parallel Supplementary Code directory, which defines and evaluates the aforementioned equations.
