# Supplementary material for: Maternal transmission as a microbial symbiont sieve, and the absence of lactation in male mammals
Source: Nat Commun. 2024 Jun 27;15:5341. doi: 10.1038/s41467-024-49559-5 (PMC11211401; doi:10.1038/s41467-024-49559-5)
Supplement: Supplementary file 6 — Reporting Summary [file 41467_2024_49559_MOESM6_ESM.pdf]

Corresponding author(s): Brennen Fagan

Last updated by author(s): Apr 25, 2024

Reporting Summary

Nature Portfolio wishes to improve the reproducibility of the work that we publish. This form provides structure for consistency and transparency in reporting. For further information on Nature Portfolio policies, see our Editorial Policies and the Editorial Policy Checklist.

Statistics

For all statistical analyses, confirm that the following items are present in the figure legend, table legend, main text, or Methods section.

n/a	Confirmed	
☐	☒	The exact sample size (n) for each experimental group/condition, given as a discrete number and unit of measurement
☐	☒	A statement on whether measurements were taken from distinct samples or whether the same sample was measured repeatedly
☒	☐	The statistical test(s) used AND whether they are one- or two-sided<br>*Only common tests should be described solely by name; describe more complex techniques in the Methods section.*
☒	☐	A description of all covariates tested
☐	☒	A description of any assumptions or corrections, such as tests of normality and adjustment for multiple comparisons
☐	☒	A full description of the statistical parameters including central tendency (e.g. means) or other basic estimates (e.g. regression coefficient) AND variation (e.g. standard deviation) or associated estimates of uncertainty (e.g. confidence intervals)
☒	☐	For null hypothesis testing, the test statistic (e.g. F, t, r) with confidence intervals, effect sizes, degrees of freedom and P value noted<br>*Give P values as exact values whenever suitable.*
☒	☐	For Bayesian analysis, information on the choice of priors and Markov chain Monte Carlo settings
☒	☐	For hierarchical and complex designs, identification of the appropriate level for tests and full reporting of outcomes
☒	☐	Estimates of effect sizes (e.g. Cohen's d, Pearson's r), indicating how they were calculated

Our web collection on statistics for biologists contains articles on many of the points above.

Software and code

Policy information about availability of computer code

Data collection	Simulations were implemented in C (standard: C11, compiler: gcc 7.5.0), as described in the methods of the main text (Stochastic birth-death model), and are available via Github available at https://github.com/Brennen-Fagan/Maternal-Transmission.
Data analysis	Analysis was conducted by hand and via Mathematica (Version: 12.0.0.0 and 12.1.1.0), as described in the methods of the main text (Algebraic model and Differential equation model), and are available via Github at https://github.com/Brennen-Fagan/Maternal-Transmission.

For manuscripts utilizing custom algorithms or software that are central to the research but not yet described in published literature, software must be made available to editors and reviewers. We strongly encourage code deposition in a community repository (e.g. GitHub). See the Nature Portfolio guidelines for submitting code & software for further information.

Data

Policy information about availability of data

All manuscripts must include a data availability statement. This statement should provide the following information, where applicable:

- Accession codes, unique identifiers, or web links for publicly available datasets
- A description of any restrictions on data availability
- For clinical datasets or third party data, please ensure that the statement adheres to our policy

Data generated from the archived code and the archived code are available at https://doi.org/10.6084/m9.figshare.22816529. These source data, used to generate the images, are provided with this paper as well.

Research involving human participants, their data, or biological material

Policy information about studies with human participants or human data. See also policy information about sex, gender (identity/presentation), and sexual orientation and race, ethnicity and racism.

Reporting on sex and gender	Not collected
Reporting on race, ethnicity, or other socially relevant groupings	See above
Population characteristics	See above
Recruitment	See above
Ethics oversight	See above

Note that full information on the approval of the study protocol must also be provided in the manuscript.

Field-specific reporting

Please select the one below that is the best fit for your research. If you are not sure, read the appropriate sections before making your selection.

☐ Life sciences ☐ Behavioural & social sciences ☒ Ecological, evolutionary & environmental sciences

For a reference copy of the document with all sections, see nature.com/documents/nr-reporting-summary-flat.pdf

Ecological, evolutionary & environmental sciences study design

All studies must disclose on these points even when the disclosure is negative.

Study description	Independent (Gillespie) simulation runs of host-symbiont systems of increasing complexity which result in either fixation or elimination of a newly introduced symbiont community, beginning with a stochastic birth-death process and adding selection for a male transmission gene and horizontal transmission of the new symbiont community. We demonstrate the symbiont sieve with 5000 realisations
Research sample	The research samples are realisations of the aforementioned simulation runs, meant to represent a generic (host-symbiont) system undergoing the symbiont sieve.
Sampling strategy	No sample-size calculation was performed. Instead, a derivation was carried out to obtain the macroscopic model of ordinary differential equations corresponding to the stochastic process (see Methods). This deterministic model describes the mean behaviour of the stochastic process. Good agreement was observed between the two methods. Parameters and initial population sizes of the simulations were chosen to have an initial equilibrium population of 1000 to avoid finite size effects (e.g. https://doi.org/10.1103/PhysRevE.70.041902). Additionally a normal distribution was postulated for the effect of novel symbiont on the host fitness, and this distribution was sampled from using standard computational methods (see Numerical Recipes in C 2nd Edition, Equation 7.2.10).
Data collection	Richard Law created the C code, performed the simulations, and recorded and reported the simulation results, but the code was checked by all authors.
Timing and spatial scale	Timing not applicable, spatial scale confined to a population of 1,000 individuals initially, but the results should generally hold for other starting population sizes.
Data exclusions	No data exclusions.
Reproducibility	The study is completely simulated and replication code is made available.
Randomization	For each simulation and its corresponding initial condition, novel symbionts were allocated uniformly at randomly among the population. Each step of the process proceeded uniformly at randomly among the appropriate (sub) population (e.g., sexual reproduction).
Blinding	Blinding was not relevant. Much of the analysis and many of the images are proof of concept of examples, and that which is not has no decision making (i.e., Figure 2 simply plots the results with and without the symbiont sieve).

Did the study involve field work? ☐ Yes ☒ No

Reporting for specific materials, systems and methods

We require information from authors about some types of materials, experimental systems and methods used in many studies. Here, indicate whether each material, system or method listed is relevant to your study. If you are not sure if a list item applies to your research, read the appropriate section before selecting a response.

Materials & experimental systems

n/a	Involved in the study
☒	☐ Antibodies
☒	☐ Eukaryotic cell lines
☒	☐ Palaeontology and archaeology
☒	☐ Animals and other organisms
☒	☐ Clinical data
☒	☐ Dual use research of concern
☒	☐ Plants

Methods

n/a	Involved in the study
☒	☐ ChIP-seq
☒	☐ Flow cytometry
☒	☐ MRI-based neuroimaging

Plants

Seed stocks	NA (The new reporting summary file seems to have the Plants check boxes above connected to Dual use research of concern.)
Novel plant genotypes	NA
Authentication	NA
